# Seasonal Shifts in the Microbiota of Wild-Caught Danish *Carcinus maenas*

**DOI:** 10.3390/microorganisms14061187

**Published:** 2026-05-25

**Authors:** Lorenzo Chinellato, Lisbeth Truelstrup Hansen, Martin L. Kragh, Nina Gringer, Claus H. Bang-Berthelsen

**Affiliations:** 1 Research Group for Microbial Biotechnology and Biorefining, National Food Institute, Technical University of Denmark, 2800 Kongens Lyngby, Denmark; claban@food.dtu.dk; 2 Research Group for Food Microbiology and Hygiene, National Food Institute, Technical University of Denmark, 2800 Kongens Lyngby, Denmark; litr@food.dtu.dk (L.T.H.); malak@food.dtu.dk (M.L.K.); 3 Research Group for Food Production Engineering, National Food Institute, Technical University of Denmark, 2800 Kongens Lyngby, Denmark; ngri@food.dtu.dk

**Keywords:** green shore crab, culturomics, genomics, seawater temperature, sustainability

## Abstract

Underutilized and abundant in the Danish coastal area, *Carcinus maenas* has become a threat to the environment and to the local fisheries. In this study we investigated the microbiota and presence of microbial hazards of interest for human health in crabs caught over the period of one year, to investigate its potential for human consumption. Between 2023 and 2024, four seasonal samples of live specimens (*n* = 5) were caught off the Lillebælt (DK) coastal area. To characterize the microbiota of the crabs, a culture-dependent approach was used to determine total aerobic mesophilic count, total aerobic psychrotrophic count, *Bacillus* spp., *Enterobacteriaceae*, lactic acid bacteria, fungi, *Salmonella* spp., *Listeria monocytogenes* and *Bacillus cereus*. MALDI-TOF was used to corroborate results and further identify isolated microorganisms. The results were then compared with data obtained from amplicon sequencing of community 16S rRNA genes to compare family-level compositions of the microbiota. Of the pathogens of interest, *B. cereus* was detected during summer/autumn, reaching a maximum of 2.5 log cfu/g. *Salmonella* spp., and *L. monocytogenes* were below the limit of detection (<1 cfu/0.1 g). Spoilage bacteria were detected (e.g., *Brochothrix* spp., *Carnobacterium* spp., *Photobacterium* spp., *Pseudomonas* spp., *Psychrobacter* spp. and *Shewanella* spp.). The study highlighted significant seasonal changes (PERMANOVA, FDR-adjusted *p* = 0.00003) in the microbial composition. The gathered evidence suggests that with proper handling, the crabs could represent a safe resource.

## 1. Introduction

The global population is expected to reach 9.8 billion by 2050 [1], intensifying the demand for nutrient-rich, sustainable, secure and safe food sources. The current food system relies on unsustainable practices; both agriculture and traditional fisheries rely on insufficient resources [2]. Such intensive exploitation causes environmental degradation and fails to allow sufficient time for ecosystem recovery.

Among various nutrient sources, aquatic animals contribute to a large share of our nutrition. According to the FAO’s report from 2024 [3], up to 15% of the animal proteins consumed were of aquatic origin. Other than proteins, seafood is also responsible for providing n-3 polyunsaturated fatty acids, vitamins and minerals essential to human nutrition [4]. Due to climate change, habitat degradation and poor fishing policies, the yields of major seafood resources are declining. The disappearance of a food source might destabilize the food system, depleting already limited food sources and threatening food security for the global population.

Diversifying the global consumption of seafood might be a solution to reduce the stress on available marine resources. Targeting underexploited invasive or overabundant species could provide a secure nutrient alternative to the current uncertainty of seafood options.

A potential candidate is *Carcinus maenas* (green shore crab or green crab). Autochthonous to Northern Europe, the green crab is commonly found in Northern Europe coastal areas as well as along the coastlines of South Africa, North and South America, where it is considered an invasive species [5,6]. The green crab’s ability to prosper in diverse habitats, from intertidal areas to the open sea, is directly linked to its omnivorous diet, rapid reproduction and adaptability to varying salinities, temperature and oxygen levels [6,7,8,9,10].

In Denmark, the ecological impact of the crab’s activity is severe. Climate change and intensive fishing of predatorial species have created hospitable conditions for *C. maenas*. The rise in the population of the green crab has become a major concern for the safeguarding of biodiversity and well-being of local fisheries in several countries [11,12,13]. The crabs’ activity damages economically valuable species and disrupts eelgrass beds, a type of marine plant exploited as an underwater nursery by numerous species, thus affecting the stability of coastal ecosystems [14]. Introducing a dedicated fishery to remove the crab from the Danish coastal areas might control their population, mitigate environmental degradation and provide a new seafood alternative to overexploited species such as cod [15]. In line with this, Duncombe et al. (2017) [16] evaluated the effect of trapping to control a population of green crab in British Columbia, showing a shift in population structure toward smaller and younger specimens following trapping efforts. While the population size remained relatively stable, the reduction in body size may have repercussions on reproductive capacity. Primary analysis of the crab’s nutrient composition suggests that the species is rich in protein (12.3% of wet weight) and contains all the essential amino acids [17], highlighting its potential as a food source. Its protein composition and high ash content (16.5% of wet weight) makes it also appealing as a potential fishmeal replacement for ash-tolerant fish species (e.g., *Gadus morhua*) or as a supplement in chicken feed [18].

Characterizing the microbial community associated with the freshly caught crabs provides an insight regarding possible pathogens and spoilage microorganisms present in the resource [19,20]. This knowledge will be essential when developing appropriate and sustainable post-harvest handling and preservation protocols and novel products. To acquire comprehensive insight into the microbiota of a given food, a combination of culture-dependent and culture-independent approaches are commonly applied [20,21].

Since little is known of the microbial communities harbored by this species of crab, the aim of this study is to investigate the microbiota of the green crabs. As part of the KRABFISK project, whose goal is to investigate the potential harvest and food/feed applications of the green crab, the accumulated evidence will form the foundation for the design of safe, sustainable and cost-effective preservation methods to store the biomass on site.

To account for potential seasonal variations in the crab’s microbiota, samples were collected at four time points over the course of one year, with three-month intervals between each sampling event.

Culture-dependent quantification and identification of viable microbes (culturomics) were compared to culture-independent characterization of the bacterial community by 16S rRNA amplicon sequencing of the bacterial community. Our study will corroborate and expand our understanding of *C. maenas*’s microbiota and its potential changes throughout a year.

## 2. Materials and Methods

### 2.1. Retrieval and Processing of Carcinus maeans

Live specimens of *Carcinus maenas* were caught utilizing lobster traps in the Kolding Fjord at an approximate water depth of 1.5 m. The animals were transferred to Fredericia (55.52568, 9.69317, Denmark, DK), where they were collected by the DTU team and transported in cooled containers within the same sampling day to the laboratory at DTU in Kgs. Lyngby (DK). The temperature was kept around 4 °C for the duration of the travel. Four samplings were performed once every 3 months between 2023 and 2024, i.e., in August (2023), October (2023), January (2024) and April (2024). Data of the water temperature during sampling was provided by DTU AQUA. During each sampling, 1 kg of crab was retrieved, and a representative number (*n* = 5) of specimens was randomly selected from the batch. The weight and sex of the animals were recorded and then processed within one hour from arrival. Each individual specimen was submerged in a volume of physiological salt peptone (FKP), at a 1:2 *w*/*w* crab to FKP ratio, and mechanically homogenized utilizing a sterile blender for 1 min. The homogenized mixture was transferred to a sterile stomacher bag with a filter (Interscience, Paris, France) to remove the suspended solids, and 1 mL of the filtered supernatant was taken for appropriate serial dilutions (until 10^−7^ and subsequent plating on agar plates. For each specimen, a 15 mL aliquot of the filtered supernatant was collected and stored at −20 °C until DNA extraction.

### 2.2. Enumeration and Detection

Aliquots of 100 μL were taken from relevant dilutions and spread on specific media: Tryptic Soy agar (TSA; SSI DIAGNOSTICA GROUP, Hillerød, Denmark) for enumeration of the total mesophilic bacteria, Long and Hammer agar (LH; SSI DIAGNOSTICA GROUP, Hillerød, Denmark) to determine total aerobic psychrotrophic bacteria count, Mannitol egg Yolk Polymyxin agar (MYP; SSI DIAGNOSTICA GROUP, Hillerød, Denmark) to assess the presumptive number of *Bacillus* spp., MacConkey agar (McC; SSI DIAGNOSTICA GROUP, Hillerød, Denmark) to estimate the presumptive counts of *Enterobacteriaceae*, De Man–Rogosa–Sharpe agar (MRS; Thermo Fisher Scientific, Waltham, MA, USA) and Modified LAB Selective agar (MLS), prepared as described by Xiao et al. 2024 [22], for the enumeration of lactic acid bacteria (LAB), Yeast Extract Peptone Dextrose agar to quantify the number of yeasts and molds, and Rapid Bacillus cereus agar (RBC; BIO RAD, Hercules, CA, USA)to provide a presumptive enumeration of *Bacillus cereus*. After the incubation, all media were visually inspected, and plates containing between 30 and 300 colony-forming units (cfu) were enumerated and recorded. Incubation time and temperature applied for each medium are reported in the Supplementary Materials, Table S1.

Detection of *Salmonella* spp. in the samples was determined by enrichment of 1 mL of the filtered crab suspension (1:10 dilution) in 9 mL of Buffered Peptone Water (BPW; 37 °C 16–20 h; Thermo Fisher Scientific, Waltham, MA, USA), followed by streaking on Xylose Lysine Deoxycholate Agar (XLD; 37 °C 16–20 h; SSI DIAGNOSTICA GROUP, Hillerød, Denmark). Presence of *Listeria monocytogenes* in the samples was determined by preliminary enrichment of a 1 mL aliquot of the filtered crab suspension in 9 mL of half-Fraser broth for 24 h at 30 °C, followed by a secondary enrichment in Fraser broth at 37 °C for 24 h (Thermo Fisher Scientific, Waltham, MA, USA). After each enrichment step was done incubating, volumes of 10 μL were streaked on Palcam (PAL; SSI DIAGNOSTICA GROUP, Hillerød, Denmark) agar plates and incubated at 37 °C for 48 h. For the detection of *Salmonella* spp. and *L. monocytogenes*, all XLD and PAL plates, respectively, were visually inspected, and presumptive positives (typical black colonies) were recorded. All plate count data were log-transformed prior to any statistical analysis.

### 2.3. Isolation

Once enumerated, the plates that displayed distinct colonies, with 30 to 300 cfu, were selected for further isolation and identification. Ten (*n* = 10) colonies were randomly selected for non-selective media (TSA, LH), while five (*n* = 5) were picked for selective agars (MYP, RBC, MRS, YPD, McC, MLS) and three (*n* = 3) for media that underwent an enrichment step (XLD, PAL). Colonies from TSA, LH, MRS, YPD, MLS were re-streaked once on the same media until a pure culture was obtained. Isolates from MYP, RBC, McC, XLD and PAL were streaked on Blood Agar (BA). All isolates were incubated as previously described for the original media until observable colonies appeared.

### 2.4. MALDI-TOF Identification

Preliminary identification of the isolates was carried out exploiting a MALDI-TOF Biotyper^®^ mass spectrometry using the Microflex LT/SH smart benchtop system (Bruker Daltonik, Bremen, Germany). The libraries employed as references were BDAL and Filamentous Fungi (Bruker), both provided by the manufacturer. Bacteria isolated in the previous step were identified in duplicates by direct smearing on the target plate, followed by lysis of the biomass with 1 µL of 70% formic acid (Sigma-Aldrich, Inc., St. Louis, MO, USA) applied to each individual well. Once dried, we applied a matrix solution prepared as indicated by Bruker’s methods, where 10 mg of alpha-cyano-4-hydroxycinnamic acid (Sigma-Aldrich, Inc., St. Louis, MO, USA) were dissolved by vortexing for 4 min in a solution composed of 475 µL of MilliQ water, 500 µL of acetonitrile ≥99% (Sigma-Aldrich, Inc., St. Louis, MO, USA) and 25 µL of trifluoroacetic acid ≥99% (Sigma-Aldrich, Inc., St. Louis, MO, USA). Aliquots of 1 µL of the solution were applied to each well and left to dry before running the plates in the instrument. Considering our interest in genus-level identification, any sample that scored higher than 1.70 was listed as a positive result and scores below the threshold were considered as “Not identified” (No ID), as recommended by the manufacturer.

### 2.5. Long-Term Storage of Isolates

Identified isolates deemed relevant for the study and for possible future studies were grown in Luria Broth (LB, 30 °C, 48 h). An aliquot of each isolate was then stored in a final glycerol concentration of 25% at −80 °C and then added to the DTU National Food Institute Culture Collection (NFICC).

### 2.6. DNA Extraction, Sequencing and Data Processing

DNA extractions were prepared for each individual, using the 15 mL aliquot collected from the 10^−1^ dilution of the specimens (as described in Section 2.1). Aliquots were thawed and centrifuged (13,000 rpm, 10 min). The resulting pellet was used for microbial DNA extraction with the DNeasy PowerSoil Kit (Qiagen, Manchester, UK), following the manufacturer’s protocol. The purity and concentration of the sample’s DNA were assessed by Nano Drop (Thermo Scientific, Wilmington, NC, USA). Amplification and sequencing of the 16S rRNA region V3-V4 were performed by Ilumina MiSeq 300 bp paired-end sequencing (Eurofins Genomics, Ebersberg, Germany). Processing of the raw sequencing data was performed with CLC Genomics Workbench 23.0.4 (Qiagen, Aarhus, Denmark), with adapter removal and quality trimming based on a maximum error probability of 0.01 (Phred 20) to retain sequencing depth, yielding 39,723 ± 5699 reads per sample.

Taxonomic profiling of filtered reads was carried out using the CLC Microbial Genomics Module 23.0.2 (Qiagen, Aarhus, Denmark) with OTU clustering based on the Silva SSU 138.2 ribosomal RNA database (99%) reference [23]. To limit the influence of low-quality reads, incomplete taxonomic classification results and low-abundance genera possessing relative abundance below 0.005% across all samples were excluded.

### 2.7. Diversity, Richness and Evenness

The microbial diversity of the samples was assessed by determining the Shannon–Wiener [24], Simpson [25,26], CHAO1 [27], Pielou’s Evenness (J) [28] and Gini–Simpson Evenness (Ev.D) [29] indices, utilizing the following equations:

Shannon–Wiener (H′)  H′ = −∑ *pi* × ln (*pi*) (1)

where *pi* indicates the proportion of taxonomic units belonging to the *i* family.

(2) $$\mathrm{Simpson}\;(D)\;\;D=1-\sum \;(\;{ni}^{2}/N^{2})$$

where *ni* stands for the number of individuals belonging to the *i* family, divided by *N*, the total number of individuals in the dataset.

(3) $$\mathrm{CHAO}1\;\;\mathrm{CHAO}1=S_{OBS}+\frac{{{(F}_{1})}^{2}}{2(F_{2})}$$

where $S_{OBS}$ depicts the total number of observed family, while $F_{1}$ represents the number of singlets and $F_{2}$ highlights the number of doublets found in the dataset.

(4) $$J\;\;J=\frac{H^{\prime }}{ln(S_{OBS})}$$

The Pilou evenness (J) is calculated from H′ divided by the total number of species (*S_obs_*). Values range from 0 to 1, with 1 indicating all species are equally abundant.

(5) $$\mathrm{Ev}.\;D\;\;\mathrm{Ev}.\;D=\frac{D}{1-(1/(S_{OBS})}$$

The Gini–Simpson Evenness uses D and S_OBS_ to express evenness, with a value of 1 representing evenness while a value of 0 would represent dominance of one species.

### 2.8. Defining the Crab’s Core Microbiota

In this study, to determine which bacterial families contribute the most to *C. maenas*’s core microbiota, we decided to set the thresholds for prevalence among all samples at 95% and levels equal to or above 0.05% relative abundance. The chosen values ensure minimal sampling bias and strong ecological relevance compared to transient families that inconsistently appear across seasons and samples.

### 2.9. Statistical Analysis

The normality test (Shapiro–Wilk), Bartlett’s test, Kruskal–Wallis (KW) test, one-way ANOVA followed by Tukey’s multiple comparison test, Dunn’s multiple comparison test, Pearson correlation analysis and Spearman’s rank test of the data recorded from culture-dependent approaches were performed using GraphPad Prism version 10.0.0 for Windows (GraphPad Software, Boston, MA, USA). The determination of alpha diversity, via calculating the Shannon–Wiener, Simpson and CHAO1 indices, was performed and visualized in CLC Genomics Workbench 23.0.4 (Qiagen, Aarhus, Denmark).

The following analyses were performed in R studio version 2026.01.2 + 418 (RStudio: Integrated Development Environment for R. Posit Software, PBC, Boston, MA) using R version 4.6.0 (2026-04-24 ucrt). The vegan package was applied for multivariate community analysis, while tidyverse and base R were applied for data handling and univariate statistical tests.

Principal Coordinate Analysis (PCoA) was performed based on the Bray–Curtis dissimilarity, calculated from the abundance data matrix of both culture-dependent and independent datasets. Samples were visualized in R using the ggplot2 package using the first two principal coordinate axes. Figures generated were then combined in Inkscape 1.4.2.

Permutational multivariate analysis of variance (PERMANOVA) was performed to assess significant differences in beta diversity among different seasonal samplings. PERMANOVA was applied on family-level 16S rRNA profiles and based on the Bray–Curtis dissimilarity using the adonis2 function in the vegan R package with 99,999 permutations. To identify month-to-month changes, pairwise PERMANOVA comparisons were performed among all sampling months and adjusted using the Benjamini–Hochberg false discovery rate (FDR). Differential abundance analysis was performed to assess community-level differences. Because the abundance data was non-normally distributed, Wilcoxon rank-sum tests were applied. For each family, log_2_ fold change was calculated as the ratio of mean abundance between the 2023 and 2024 samples. Resulting *p*-values were adjusted for multiple testing using FDR. To ensure biological relevance, additional filtering combined maximum mean relative abundance above 0.1% and absolute log_2_ fold change above 1.

### 2.10. Reconciliation Analysis

Reconciliation between sequencing-based and culture-dependent datasets was assessed by the application of Jaccard similarity, based on presence–absence of bacterial families in the data [30].

## 3. Results

### 3.1. Sample Data

Specimens selected for the study had an average weight of 38.33 ± 17.80 g. After inspection, out of the 20 randomly selected animals, 65% of the population was identified as males and 35% were females. The following water temperatures were recorded for samples obtained in August 2023 (8.2023), October 2023 (10.2023), January 2024 (1.2024) and April 2024 (4.2024): 16.1, 12.5, 2.5 and 6.5 °C.

### 3.2. Culture Dependent

#### 3.2.1. Enumeration

The summary of the microbial count results obtained from each seasonal sampling (8.2023, 10.2023, 1.2024 and 4.2024) is depicted in Table 1.

The total aerobic mesophilic count on TSA ranged from a maximum of 6.07 ± 1.70 log cfu/g during the 8.2023 sampling to a minimum of 4.85 ± 0.70 log cfu/g in 4.2024 (for more information, see Table 1). Shapiro–Wilk test confirmed normality and Bartlett’s test highlighted violation of homoscedasticity (*p* = 0.0038); therefore, WelcH′s ANOVA test was performed and confirmed no significant differences (*p* > 0.05).

The total aerobic psychrotrophic count was determined using LH. We observed the highest count of 5.67 ± 0.31 log cfu/g in autumn (10.2023). Throughout the whole year, the value remained stable, approaching its lowest level in spring (4.2024) 5.28 ± 0.65 log cfu/g. Normality was confirmed by the Shapiro–Wilk test, and homogeneity of variance was evaluated by Bartlett’s test (*p* = 0.1974). ANOVA indicated no significant differences among group means (*p* > 0.05).

The maximum level of presumptive *Enterobacteriaceae* observed on McC agar was registered in 10.2023 with a total count of 3.69 ± 0.10 log cfu/g, while in contrast, the 8.2023 yielded no typical colonies, indicating *Enterobacteriaceae* counts below the detection limit of 2 log cfu/g. Seasonal differences were confirmed (*p* = 0.0009, Kruskal–Wallis for non-normal data).

The data for the total count of presumptive *Bacillus* spp. detected on MYP violated the normality test, and so Kruskal–Wallis was implemented, verifying significant variation (*p* = 0.0108). The highest registered value of 5.47 ± 0.91 log cfu/g was detected in autumn 2023, while all other sampling seasons scored lower values. Enumeration of presumptive *Bacillus cereus* on RBC returned low concentration throughout all seasons, with 2.48 log cfu/g as the maximum level observed in one out of five specimens in 8.2024, while the remaining four samples presented no typical colonies.

Presence of *Salmonella* spp. on XLD and *Listeria* spp. on Palcam was not detected in any of the twenty analyzed specimens. Any colony showing morphological similarities to the target organisms was isolated and identified via MALDI-TOF.

Determination of presumptive lactic acid bacteria on MRS and MLS was subject to fluctuations throughout the year, reaching the maximum on sampling 10.2023 with 4.69 ± 0.15 log cfu/g and 3.97 ± 0.28 log cfu/g, respectively. Both MRS (*p* = 0.0005) and MLS (*p* = 0.0008) show significant differences (Kruskal–Wallis), i.e., higher levels, in October 2023.

Presumptive counts of yeasts and molds registered values of 4.58 ± 0.14 log cfu/g on 10.2023; however, further identification through microscopy and MALDI-TOF confirmed the presence of bacterial contamination of the YPD agar. No further statistical analysis was performed on YPD data.

The plate count dataset showed no (*p* > 0.05) correlation with the changes in water temperature, following the testing of the relationship by Pearson correlation analysis for LH and Spearman’s rank test for TSA, McC, MYP, MLS and MRS.

#### 3.2.2. Culture Dependent Identification (MALDI-TOF)

Preliminary identification via MALDI-TOF was performed on a total of 1060 purified isolates, identifying 49 genera belonging to 24 families across all isolates, including 14 families from non-selective media.

**Figure 1:**
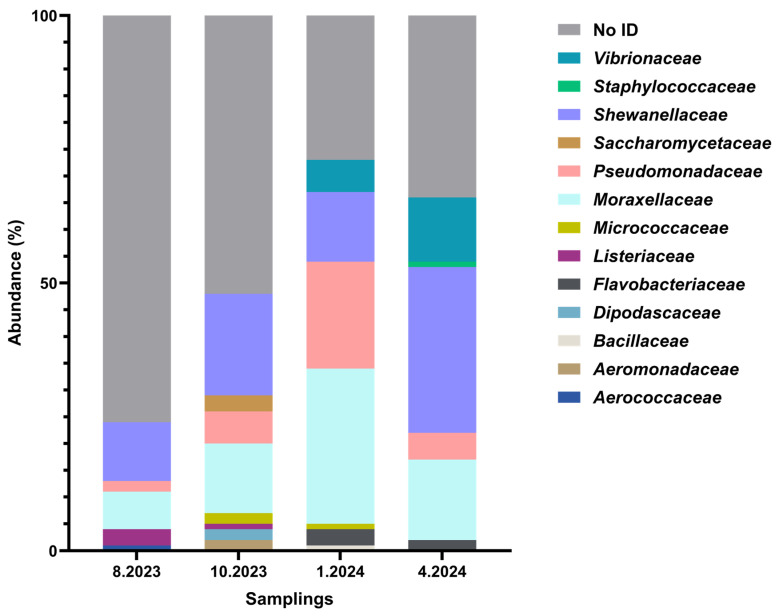
Seasonal abundance of bacterial families from non-selective media (LH and TSA) identified via MALDI-TOF.

In Figure 1, we can observe that each sampling season was characterized by the presence of a high degree of Not possible to identify (No ID) isolates. Sampling season 8.2023 scored highest with 76% unknown isolates, while the average score for other seasons approached 30%. The database produced from the analysis of the four sampling seasons, as depicted in Table 2, confirmed the presence of *B. cereus* in crabs harvested during three of the seasons (8.2023, 1.2024 and 4.2024) but not in the fall (10.2023). The presumptive *B. cereus* colonies were all isolated from RBC plates. Isolates belonging to *Enterobacteriaceae* were found during all sampling seasons. As seen in Table 2, no other matches for pathogenic bacteria of interest emerged from the identification of the purified isolates.

Correlation analysis (Spearman’s correlation rank test) of the collected culture-based dataset did not show a clear relationship with the changes in water temperature in the Kolding Fjord.

A compendium of the species identified by MALDI-TOF is available in Table S3 found in the Supplementary Materials. Several genera, including *Bacillus* spp., *Brochothrix* spp., *Pseudomonas* spp., *Shewanella* spp. and *Staphylococcus* spp., were recorded across multiple seasons. In contrast, other genera were intermittently detected. For instance, genera usually associated with marine environment and seafood, like *Vibrio* spp., *Aliivibrio* spp., *Photobacterium* spp. (all *Vibrionaceae*) and *Psychrobacter* spp. (*Moraxellaceae*), were recurrently observed. The data is recorded as presence or absence, since microorganisms were isolated from both selective and non-selective agars.

#### 3.2.3. NGS 16S RNA

**Figure 2:**
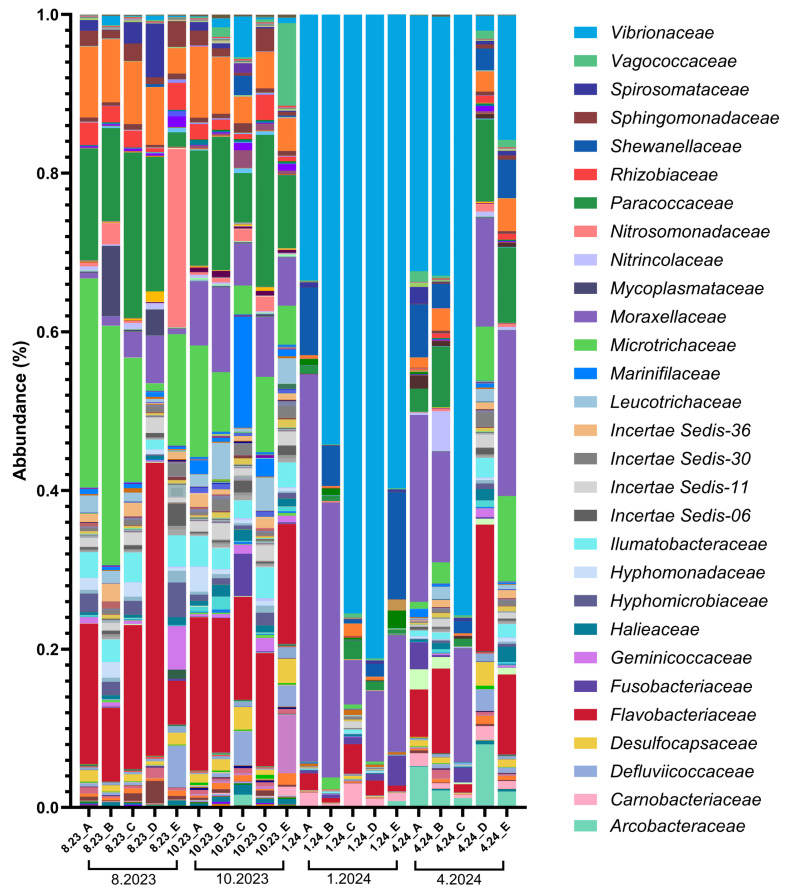
Family-level representation of bacterial community seasonal variability observed from NGS 16S rRNA results. Seasonal samples 8.2023 (summer), 10.2023 (fall), 1.2024 (winter) and 4.2024 (spring) are divided into five selected crab specimens (A–E). Legend displays 30 most abundant families present across samples.

Next Generation Sequencing (NGS) analysis was performed to characterize the microbial communities. This provided detailed taxonomic profiles and relative abundances, which were then used to compute the diversity metrics. From the analysis of the 794,463 16S rRNA OTUs, a total of 170 families were identified from the crab samples based on classification of an average of 39,723 ± 5699 reads for each sample. In Figure 2, the 30 most abundant families, which contribute to the crab microbial community for each sample, are reported.

The statistical analysis of the microbiota revealed that most families (146/170) showed significant (Kruskal–Wallis, *p* < 0.05) changes across sampling seasons. *Flavobacteriaceae*, which were dominant (17.5% ± 10.8%) during 8.2023 and autumn (15% ± 2.23%), drastically decreased during 1.2024 (1.18% ± 1.15%) (10.2023–1.2024, *p* = 0.0166). In the summer samples, *Microtrichaceae* (17.4% ± 10.3%) and *Paracoccaceae* (13.1% ± 6.4%) were also among the dominant families, but declined during winter (*Microtrichaceae* 0.5% ± 0.5, *p* = 0.008; *Paracoccaceae* 1.13% ± 0.67%, *p* = 0.0234).

The *Vibrionaceae* family displayed low values during the summer–autumn period (8.2023, 0.4% ± 0.4%; 10.2023, 1.5% ± 1.9%), while exhibiting the highest abundance among bacterial families in the winter sample (60.8% ± 16.9%). The largest seasonal increase in *Vibrionaceae* occurred between the summer and winter samples (8.2023–1.2024, *p* = 0.0046). Similarly, the dataset highlighted the increase in *Moraxellaceae*, from 2.4% ± 2.1% during 8.2023, to 22.51% ± 16.56% observed in winter 1.2024 (8.2023–1.2024, *p* = 0.0197).

Unlike Section 3.2.2, the culture-independent approach failed to highlight presence in the samples of both *Enterobacteriaceae* and *Bacillaceae*. Meanwhile, it revealed presence of *Clostridiaceae* in (0.43% ± 0.73%) most samples.

Despite the observed seasonal changes in microbial community composition, Spearman’s rank correlation test indicated no significant (*p* > 0.05) association between the relative abundance of any bacterial family and water temperature variation.

#### 3.2.4. Analysis of Diversity

**Figure 3:**
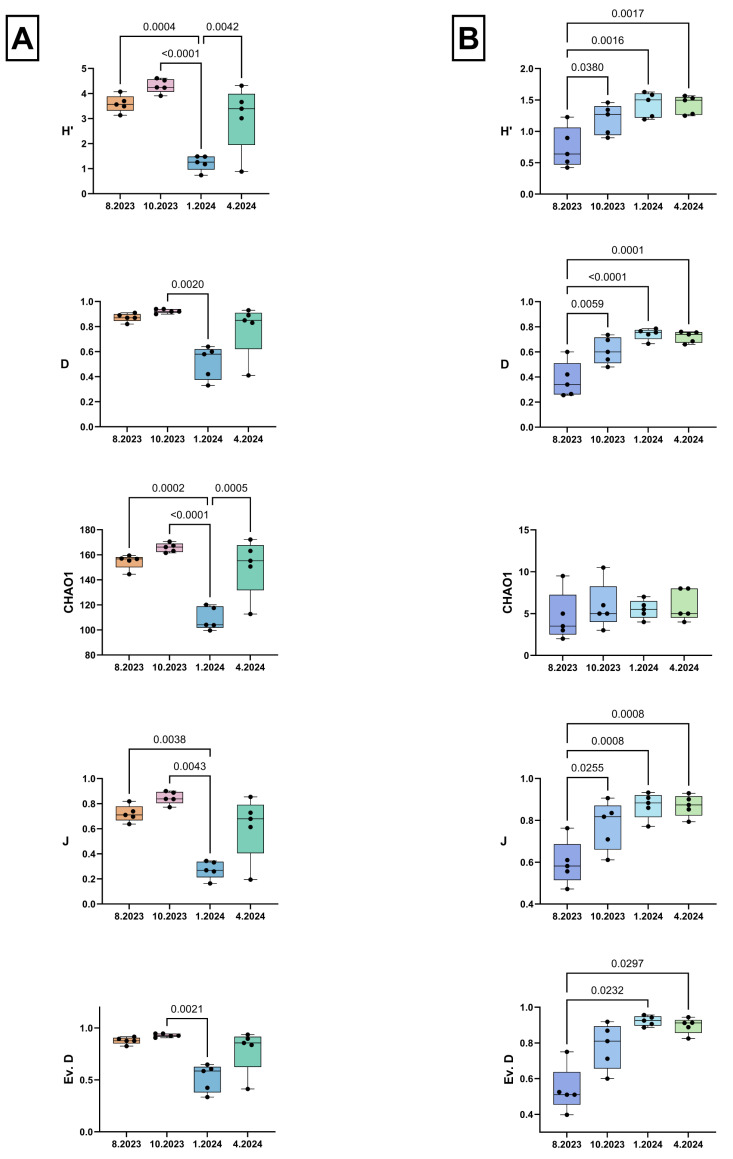
Visual comparison of indices H′, D (diversity), CHAO1 (richness), J and Ev.D (evenness) calculated for each seasonal sampling (8.2023 to 4.2024). In panel (**A**), indices were calculated based on 16S rRNA sequencing dataset; panel (**B**) indices were determined from culturomics dataset.

Seasonal variability in the microbiota was investigated by calculating alpha diversity, applying two diversity indices, a richness index, and two evenness indices: Shannon–Wiener (H′), Simpson (D), CHAO1, Pielou (J) and Gini–Simpson (Ev.D) (Figure 3). Results depended on the databases used to determine the biodiversity indices, namely either the 16S rRNA sequencing-based (panel A) or culture-based database, using isolates from non-selective media (TSA, LH) identified by MALDI-TOF (panel B), with limited agreement between the two approaches. For example, the sequencing-based analysis showed evenness indices, like J and Ev.D, were lowest in 1.2024, where a restricted number of bacterial families were dominant in the samples (e.g., *Vibrionaceae* and *Moraxellaceae*) (Figure 3A). In contrast, Figure 3B shows H′, D, J and Ev.D increasing until 1.2024. For the culture-based approach, no significant (*p* > 0.05) changes were found for CHAO1, while the sequencing-based results showed significant (*p* < 0.05) seasonal differences among seasons. As shown in Figure 3A, determination of alpha diversity based on Shannon entropy, comparing samplings across seasons showed significant variation at the family level between seasons 8.2023–1.2024, 10.2023–1.2024 and finally 1.2024–4.2024 (Tukey’s *p* < 0.05). H′ underlined higher values of alpha diversity during 8.2023 and 10.2023, while it decreased in 1.2024 and recovered in 4.2024. Simpson’s index and CHAO1 mirrored H′, emphasizing the decrease in diversity of the samples during winter and spring months. Meanwhile, the culture-based data showed significant (*p* < 0.05) shifts in H′ and D with values tending to be lower for 8.2023–10.2023 compared to 1.2024 and 4.2024.

**Figure 4:**
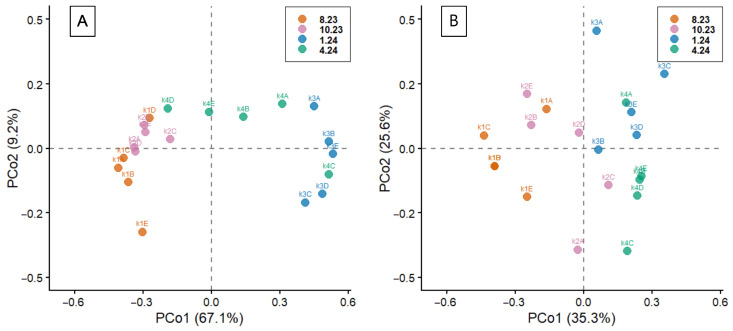
PCoA based on beta diversity of four seasonal groups, colored and labeled based on sampling month (8.2023: 16.1 °C, 10.2023: 12.5 °C, 1.2024: 2.5 °C and 4.2024: 6.5 °C). Depicted in panel (**A**), PCoA is based on 16S rRNA dataset, while in panel (**B**), PCoA is based on MALDI-TOF identifications.

In Figure 4A, the Principal Coordinate Analysis (PCoA) from the 16S rRNA dataset, based on the Bray–Curtis dissimilarity, displayed presence of outliers and within-group widely dispersed points. PCo1 explained 67.11% of the total variation. When considering the data points as half-yearly seasons, summer–autumn and winter–spring, partial clustering (January–April versus August–October) along the PCo1 axis can be observed. Samples belonging to 2023 are found in the negative section of the PCo1 axis, while samples from 2024 were predominantly distributed on the positive side of PCo1. A similar distribution was also observed when PCoA was performed on the culture-dependent dataset. In Figure 4B, PCo1 explained 35.3% of total variation, again with 2023 samples distributed in the negative section of the PCo1 and 2024 samples spread on the positive section of the axis. Both culture-dependent and independent approaches suggest a half-yearly shift in bacterial community composition.

The results of a PERMANOVA with FDR adjustment were used to determine the effect of season in community composition, as presented in Table S2 for both culture independent and culture-dependent datasets. Global PERMANOVA applied to 16S rRNA dataset revealed statistically significant differences among the tested months (R^2^ = 0.65171, FDR-adjusted *p* = 0.00001). As observable in Table S2, section A, pairwise comparison highlighted significant differences between samples 8.2023 and 1.2024 (FDR-adjusted *p* = 0.01236) and 4.2024 (FDR-adjusted *p* = 0.01236). Similarly, 10.2023 exhibited significant differences between 1.2023 and 4.2024 (FDR-adjusted *p* = 0.01236). Although marginal, when compared, 1.2024 and 4.2024 showed differences (FDR-adjusted *p* = 0.04922). Finally, pairwise comparison of 8.2023–10.2023 did not reveal statistically significant variation in community composition.

When grouped in half-yearly seasons, 8.2023–10.2023 and 1.2024–4.2024 show significant differences (FDR-adjusted *p* = 0.00003). As depicted in Figure S1, differential abundance analysis between half seasons indicates that 120 out of 170 bacterial families underwent significant changes (FDR-adjusted *p* < 0.05) between summer–autumn 2023 and winter–spring 2024. A total of 113 families exhibited negative log_2_ fold changes, 50 of which with an absolute value ≥1, indicating that these families were at least two-fold more abundant in summer–autumn compared to winter–spring samples. Meanwhile, in the 2024 samples, only seven families were subjected to significant changes, among which were *Vibrionaceae* (log_2_ = 5.64, FDR-adjusted *p* = 0.004), *Shewanellaceae* (log_2_ = 3.95, FDR-adjusted *p* = 0.004), *Psychromonadaceae* (log_2_ = 8.47, FDR-adjusted *p* = 0.004), *Pseudomonadaceae* (log_2_ = 3.19, FDR-adjusted *p* = 0.023) and *Moraxellaceae* (log_2_ = 2.00, FDR-adjusted *p* = 0.005). The differential abundance analysis of the sequencing data corroborated the hypotheses of a seasonal shift in the crab’s microbial community. In contrast to the 16S rRNA dataset, PERMANOVA applied to the MALDI-TOF data did not reveal significant changes in bacterial community composition (R^2^ = 0.003, *p* = 0.979).

#### 3.2.5. Core Microbiota Characterization

In accordance with the definition of core microbiota set in this study, when applied to the culture-independent dataset, eight families met the criteria. While the relative abundance fluctuated across the four seasons, the eight families *Vibrionaceae* (23.57% ± 29.07%), *Flavobacteriaceae* (10.95% ± 8.7%), *Moraxellaceae* (12.42% ± 11.72%), *Paracoccaceae* (8.41% ± 6.71%), *Saprospiraceae* (3.64% ± 3.07%), *Ilumatobacteraceae* (1.86% ± 1.44%), *Desulfocapsaceae* (1.03% ± 0.94%) and *Vagococcaceae* (0.9% ± 2.21%) remained consistently present throughout the year, indicating their role as important members of the microbiota in these samples.

#### 3.2.6. Reconciliation of Culture-Dependent and Independent Datasets

Reconciliation analysis assessed by Jaccard similarity between the 16S rRNA and the MALDI-TOF datasets displayed low values (0.0316 ± 0.0151). The determined values highlight the limited overlap in microbial community composition of the two datasets.

## 4. Discussion

In this study, we aimed to analyze the bacterial community of *C. maenas* by coupling culturomics and sequencing-based methods to determine the composition of the indigenous microbiota as well as the presence of human bacterial pathogens in specimens obtained throughout the year.

The microbial composition of the green crab observed in this study showed concordance with the results obtained by Koepper et al. (2023) on the invasive green crab’s microbiota [31]. Although the animals belong to different geographical regions, both of our studies consistently identified *Flavobacteriaceae*, *Alphaprotebacteria* (*Paracoccaceae*) and *Gammaprotebacteria* (*Vibrionaceae* and *Moraxellaceae*) as dominant in the crab’s microbial community. Notably, the prevalence of *Flavobacteriaceae* and Alphaproteobacteria in the 2023 August and October samples mirrors the seasonal window examined by Koepper et al., whose sampling was in summer–autumn 2018. The *Alphaprotebacteria* and *Gammaprotebacteria* present are also among the classes of bacteria described by Rusanova et al. (2025) [32] regarding the composition of the Norway lobster’s (*Nephrops norvegicus*) microbiota collected from different geographical areas. The reoccurrence of these taxa across different species and regions suggests that they represent crustacean-associated groups.

Of the families found in the crab samples, *Moraxellaceae* and *Flavobacteriaceae* are commonly associated with chitinoclastic bacteria, which are among the main causes of shell disease in crustaceans [33]. In the case of *Vibrionaceae*, the family may be found as commensals in healthy animals, for instance on the animal’s exoskeleton, but if present in the haemolymph of the crab, the bacteria may cause bacteremia, weaken the crabs and increase the risk of mortality [34]. *Vibrionaceae* are commonly found in marine environments usually associated with higher water temperatures [35,36,37,38]. In our study, contrary to what has been reported in the literature, both culturomics and 16S rRNA sequencing results highlighted an increase in the relative abundance of *Vibrionaceae* in the autumn and winter samples. This phenomenon could be explained by the increase in psychrotrophic *Vibrionaceae* species, e.g., *Photobacterium* spp. and *Aliivibrio* spp. [39,40] during colder months. As observable in Table S3, the MALDI-TOF analysis of bacterial isolates collected in this study displayed presence of *Photobacterium iliopiscarium* and *Aliivibrio sifiae* in 2024 winter and spring samples. In their study exploring potential reservoirs of *Vibrio* spp. during the winter period, Möller et al. (2021) [41], however, indicated that the genus *Photobacterium* might introduce false positives in the identification of *Vibrio* spp. through 16S rRNA sequencing, potentially biasing the relative abundances at genus and family levels.

An aspect to be considered is the presence of bacteria associated with food spoilage and their seasonal abundance in the crabs. As defined by Snyder et al. (2024) [42], microbial spoilage might present itself as multiple defects affecting the organoleptic profile of the raw material (e.g., odor, taste, color and texture) and a variety of bacteria can be responsible for this phenomenon. Among the bacteria responsible for the deterioration of marine resources, *Brochothrix* spp., *Carnobacterium* spp., *Photobacterium* spp., *Pseudomonas* spp., *Psychrobacter* spp. and *Shewanella* spp. are genera that have been reported to reduce the quality in crustaceans and other seafoods [43,44,45]. By combining NGS and culture-dependent data, we were able to verify the presence of all six genera throughout the four seasonal samplings. *Shewanella* spp., *Pseudomonas* spp., and *Psychrobacter* spp. were detected at all time points and by both methods. These genera were most abundant during the winter–spring period. *Carnobacterium* spp. was observed at all time points according to the NGS data but was not detected on any growth media in 1.2024 (see Table S3). Similarly, 16S rRNA sequencing found *Photobacterium* spp. to be present in all samples, but in culture-based analysis, the bacterium was identified only in samples from April 2024. The data regarding *Brochothrix* spp. is inconsistent. According to the sequencing approach, the genus is missing in 4.2024 samples; however, we were able to isolate and identify colonies of *Brochothrix* spp. in crabs from all sampling points. Members of *Enterobacteriaceae*, which can also cause spoilage, were detected in samples from October and January. Although presence of these bacteria is not necessarily proof of causation of spoilage in the green crab, understanding how the presence of these genera is affected by seasonality, could improve the optimization of preservation strategies targeting inhibition of these spoilage bacteria to reduce post-harvest quality loss of the green crab.

Looking at the effect of temperature on the composition of the microbial community between October and January, when the water of the fjord dropped below 10 °C, we observed that the relative abundance of various bacterial families, including *Flavobacteriaceae* and *Microtrichaceae*, dropped, while *Moraxellaceae* and *Vibrionaceae* became dominant. Although the statistical analysis of our datasets confirmed strong seasonal shifts in the crab’s microbiota, these alterations could not be attributed to the change in sea-water temperature alone. The composition of the crab microbiota may also depend on the habitat and potential factors influencing it. Shifts in environmental conditions such as salinity, oxygen content, available nutrients and temperature of the water might be the leading drivers of the change in the composition of the crab’s microbiota [46,47,48,49]. While PERMANOVA analysis highlighted significant seasonal-related differences in community composition, the interpretation of these results should consider that the analysis is influenced by effect size and sample size. Given that a limitation of the study consisted of its small sample size (*n* = 5 per group), the analysis might lack the power to detect weaker effects. Therefore, these results should be considered with caution.

When assessing microbial diversity in crabs across seasons, the results of the analysis of culture-dependent and sequencing-based data appear contradictory, as confirmed by the reconciliation analysis. For example, the diversity indexes H′, D and CHAO1 in Figure 3, culture-based panel B, highlighted 1.2024 as the month with the highest diversity value, while in the sequencing-based panel A, the same month scored lowest. An influencing factor is the resolution obtained when using a sequencing method versus a culture-dependent approach. In the culture-independent approach, a total of 170 families were detected, while the culture-dependent method identified 14.

An explanation of the encountered discrepancy might be connected to our ability to select for a variety of bacteria, which will be limited by the agar media composition, chosen growth conditions and viability of the cells. The culture-based methodologies implemented in this study were employed to characterize bacteria and fungi with relevance to food quality and safety. Non-selective agar media, such as TSA, were applied to estimate the total viable count and its composition. Meanwhile, LH was utilized to enumerate psychrotrophic and slow-growing marine bacteria. In both cases, fast-growing bacteria might overgrow fastidious and less competitive microorganisms, potentially hindering our ability to capture a representative record of the culturable bacteria via MALDI-TOF. The selective media utilized in the study were chosen to explore the quantitative (e.g., McC, MYP, RBC) or qualitative (e.g., use of PAL and XLD following enrichment) presence of potential human pathogens in *C. maenas*. Another limitation of this study was the absence of incubation under anaerobic conditions. As later highlighted by the 16S rRNA dataset, DNA from anaerobic bacterial families, such as *Clostridiaceae,* were present in the sample.

In addition, a fraction of the isolated bacteria might remain unidentified due to the limited coverage of the MALDI-TOF reference database. In this study, we utilized the standard libraries provided by Bruker, which may have affected successful identification of the bacterial isolates due to the limited availability of characterized marine and environmental bacteria in the database. Taken together, this may explain how the culture-dependent approach may lead to an underestimation of diversity.

On the other hand, the NGS data is also subjected to potential artifacts. Amplicon sequencing will consider any genetic information present within the specimens and, importantly, is unable to discriminate between viable and non-viable cells. The results might misrepresent the microbiota composition of the crabs, over-estimating the number of taxonomic units present at each given time point. Moreover, the use of universal primers in amplicon sequencing is known to result in sequencing biases, thus enriching some families disproportionately [50]. As explored by Yap et al. (2022) [51], a potential procedure utilizing propidium monoazide (PMA) to reduce the impact of signals from non-viable cells on the microbiota’s composition could provide a solution to the current bias. Although promising, no reliable protocol has been verified.

Of the investigated human pathogens, presumptive *B. cereus* was detected exclusively during the summer–autumn period, becoming undetectable in the 2024 winter and spring samples, and with the highest counts in October of 2023. During the study period, *Salmonella* spp. and *Listeria* spp. were non-detectable (below the LOD) by either culture-dependent or independent methods. Although the complete absence of *Salmonella* spp. and *Listeria* spp. from our gathered data could be interpreted as a positive result, if the green crab is to be used as a food product, the results should be interpreted with a degree of caution, given the sample size as well. However, our inability to detect any of the genera could also be explained by a low abundance in the geographical area where the samples were harvested. According to the sanitary survey reports executed on populations of blue mussels in the same geographical area of the Kolding Fjord between 2007 and 2021, *Salmonella* spp. could not be detected at any of the locations [52,53]. Also, *E. coli* concentrations were ≤230 MPN/100 g of the sample in 97% of all samples examined, indicating the limited presence of fecal pollution sources.

*B. cereus* is commonly found in soil [54], and a potential explanation for its presence in the crabs could be the microbial composition of the sediment in the fishing area. Although there are no studies to support it, another explanation could be a direct relationship between *B. cereus* and the crabs themselves. According to Margulis et al. (1998) [55], the pathogen is often found in the gut microbiota of certain arthropods, where it is attached to the gut epithelium in its filamentous form. Due to its spore-forming capacity and toxin production associated with both emetic and diarrhoeal illness [56], the presence and growth potential of *B. cereus* in the crabs must be taken into consideration. As highlighted by EFSA [57], concentration levels equal or above 5 log CFU/g have been associated with most cases of food-borne outbreaks by *B. cereus*, although some exceptions are made due to the diverse strain-dependent pathogenic capacity. Therefore, in the case of the development of a crab-based product, since *B. cereus* was detected in the raw material, it is of utmost importance that concentrations of said species are monitored periodically, both in the fresh crabs and in the final product.

While *L. monocytogenes* and *Salmonella* spp. were not detected in the present study, concentrations of human pathogens in the waters are highly susceptible to anthropogenic activities, which could cause alteration to the marine environment and its microbial communities, for instance by the release of sewage, farm manure and industrial waste into the surrounding areas [58]. The human pathogens present in these wastewaters can persist in the coastline accumulating in seawater, sediments and living organisms [59]. If we evaluate the green shore crab’s habitat predilection and benthic lifestyle, changes in the marine composition could increase the risk of exposure to such pathogens. In case of development of food products made from green crabs, it would be important to ensure that products comply with current microbiological criteria (e.g., in the EU defined by Regulations (EC) No. 2073/2005 and 1441/2007 [60]). Rapid application of post-harvest procedures is therefore needed to inhibit growth of the indigenous microbiota during subsequent storage. Refrigeration (≤4 °C) is currently the method applied to store the caught crabs directly at the harbors. However, high energy demand and infrastructural investments costs to maintain cold storage can limit feasibility for small-scale fishing operations. Future studies should therefore explore more sustainable and accessible preservation strategies, such as salting and fermentation at environmental temperatures to reduce energy dependence [61]. Besides conforming to standard hygiene and good manufacturing practices, introducing efficient preservation methods will help reduce the risk of hazardous bacteria or deterioration of the biomass during storage.

A microbial safety evaluation of the green crab’s fitness to be applied as animal feed should also be executed. Consistent with the EU Commission Regulation (EU) No. 142/2011 [62] regarding the microbial standard for feed, *Salmonella* spp. were not detected in the analyzed samples. In contrast, *Enterobacteriaceae* were detected above the regulatory limits in two out of four samplings. As anaerobic cultivation was not performed, the presence or absence of *Clostridium perfringens* was not assessed. It should also be noted that a more targeted analysis to confirm compliance with the criteria should be conducted on the final product intended to be used as animal feed. According to the downstream processing of the biomass (e.g., refrigeration, heat treatment, drying, salting or fermentation), microbial loads may substantially change.

In the present study, we examined the microbiota of industrially caught and handled *C. maenas*, which was in line with the aim to identify the microbiota to be controlled in subsequent preservation steps. Other factors, which could have been considered but were not included in this study, are the fishing method and handling procedures, which may affect the results of the microbiota analysis. The choice of bait and handling conditions of the animals could similarly introduce microbial contamination, thereby altering the native microbiota.

## 5. Conclusions

In this study, we described the microbial community composition of commercially harvested *C. maenas* for the first time, providing evidence of its seasonal shifts and dominance of *Flavobacteriaceae*, *Alphaprotebacteria* (*Paracoccaceae*) and *Gammaprotebacteria* (*Vibrionaceae* and *Moraxellaceae*) in the crab’s microbial community. Putative spoilage bacteria (*Brochothrix* spp., *Carnobacterium* spp., *Photobacterium* spp., *Pseudomonas* spp., *Psychrobacter* spp. and *Shewanella* spp.) were also detected. *B. cereus* was the only pathogen detected. *Salmonella* spp. and *Listeria* spp. were either absent in the green crabs or present at concentrations below the limit of detection (1 cfu/0.1 g) of the methods used in this study. These findings have direct implications for the use of green crabs as food or feed: the presence of potential spoilage bacteria and *B. cereus* indicates that preservation strategies are needed to minimize potential food waste and safety risks, particularly if crabs are used in processed products or feed formulations. The low abundance or absence of other major pathogens known from crustaceans suggests that, under proper handling and processing, green crabs could represent a safe resource.

Future studies should consider expanding the sampling area to investigate how environmental differences might affect the crab’s microbiota composition, as this may influence safety and quality, thereby paving the way for the sustainable use of green crabs in food and feed applications.

## Figures and Tables

**Table 1:** Microbial counts on LH, TSA, RBC, McC, PAL, XLD, MYP, MRS, MLS and YPD for 4 seasonal samplings.

					8.2023		10.2023		1-2024		420.24
Category	Media	n	LOD	Detected	log cfu/g	Detected	log cfu/g	Detected	log cfu/g	Detected	log cfu/g
Total aerobic psychrophilic count	LH	5	2 log	5/5	5.45 ± 0.88	5/5	5.67 ± 0.31	5/5	5.53 ± 0.38	5/5	5.28 ± 0.65
Total aerobic mesophilic count	TSA	5	2 log	5/5	6.07 ± 1.70	5/5	6.00 ± 0.35	5/5	5.49 ± 0.30	5/5	4.85 ± 0.70
*B. cereus*	RBC	5	2 log	1/5	2.48	1/5	2.08	0/5	<2 log	0/5	<2 log
*Enterobacteriaceae*	McC	5	2 log	0/5	<2 log	5/5	3.69 ± 0.10	5/5	3.18 ± 0.72	0/5	<2 log
*Bacillus* spp.	MYP	5	2 log	4/5	3.82 ± 1.95	5/5	5.47 ± 0.91	5/5	3.72 ± 0.30	5/5	3.21 ± 0.51
Presumptive LAB	MRS	5	2 log	0/5	<2 log	5/5	4.69 ± 0.15	0/5	<2 log	2/5	2.48–2.56
Presumptive LAB	MLS	5	2 log	0/5	<2 log	5/5	3.97 ± 0.28	1/5	<2 log	0/5	<2 log
Yeasts	YPD	5	2 log	0/5	<2 log	5/5	4.58 ± 0.14	5/5	3.23 ± 0.38	5/5	3.12 ± 0.37
*Salmonella* spp.	XLD	5	absence/presence	0/5	absent	0/5	absent	0/5	absent	0/5	absent
*Listeria* spp.	PAL	5	absence/presence	0/5	absent	0/5	absent	0/5	absent	0/5	absent

**Table 2:** Presence (+) or absence (−) of pathogens and microorganisms of interest in the harvested crabs isolated, from both selective and non-selective media, and identified by MALDI-TOF.

	8.2023	10.2023	1.2024	4.2024
*Listeria monocytogenes*	−	−	−	−
*Bacillus cereus*	+	−	+	+
*Enterobacteriaceae*	+	+	+	+
*Vibrio* spp.	+	−	+	+
*Salmonella* spp.	−	−	−	−

## Data Availability

The produced data will be shared upon direct request to the corresponding author.

## Supplementary Materials

The following supporting information can be downloaded at: https://www.mdpi.com/article/10.3390/microorganisms14061187/s1. Table S1: List of utilized media, incubation temperature and time; Table S2: Results of pairwise and overall PERMANOVA test comparing groups of each sampling month: 8.2023, 10.2023, 1.2024 and 4.2024. (A) displays results obtained from 16S rRNA data, (B) displays results obtained from culture-dependent data; Table S3: List of isolated microorganisms at species level identified by MALDI-TOF of the isolated bacteria from selective and not-selective agar media; Figure S1: Bar plot showing bacterial families exhibiting half-season differential abundance between 2023 and 2024. Bars represent log_2_ fold change for bacterial families displaying both large effect size (|log_2_| ≥ 1) and statistically significant differences (*p* < 0.05). Highlighted in blue are negative fold changes and positive in red.

## Abbreviations

The following abbreviations are used in this manuscript:

DTU	Danish Technical University
cfu	Colony-forming unit
LH	Long and Hammer agar
TSA	Tryptic Soy agar
RBC	Rapid Bacillus cereus agar
McC	MacConkey agar
MYP	Mannitol Egg Yolk Polymyxin agar
MRS	De Man–Rogosa–Sharpe agar
MLS	Modified LAB Selective
YPD	Yeast Peptone Dextrose agar
XLD	Xylose Lysine Deoxycholate agar
PAL	Polymyxin-Acriflavin-Lithium-Ceftazidime-Aesculin-Mannitol Agar
BA	Blood agar (TSA + 5% calf blood)
BPW	Buffered Peptone Water
LB	Lemuria Broth
MALDI-TOF	Matrix-assisted Laser Desorption/Ionization-Time of Flight
NFICC	National Food Institute Culture Collection
H′	Shannon–Wiener’s diversity index
D	Simpson’s diversity index
J	Pielou’s Evenness index
Ev.D	Gini–Simpson’s Evenness index
ANOVA	Analysis of Variance
PERMANOVA	Permutational multivariate analysis of variance
No ID	Not possible Identification
NGS	New Generation Sequencing
PCoA	Principal Coordinate Analysis
EFSA	European Food Safety Authority
