# Supplementary material for: Seasonal Shifts in the Microbiota of Wild-Caught Danish Carcinus maenas
Source: Microorganisms. 2026 May 25;14(6):1187. doi: 10.3390/microorganisms14061187 (PMC13302918; doi:10.3390/microorganisms14061187)
Supplement: Supplementary file 1 [file microorganisms-14-01187-s001.zip › microorganisms-4275542-supplementary.pdf]

Supplementary material

Table S1. List of utilized media, incubation temperature and time.

Agar/ Broth Media	Abbreviation	Temperature (˚C)	Time (h)	Provider
Long and Hammer agar	LH	15	168	SSI DIAGNOSTICA GROUP, Hillerød Denmark
Tryptic Soy agar	TSA	30	24	SSI DIAGNOSTICA GROUP, Hillerød Denmark
Rapid Bacillus cereus agar	RBC	30	24	BIO RAD, Hercules, California, USA
MacConkey agar	McC	37	48	SSI DIAGNOSTICA GROUP, Hillerød Denmark
Mannitol Egg Yolk Polymyxin agar	MYP	30	24	SSI DIAGNOSTICA GROUP, Hillerød Denmark
De Man–Rogosa–Sharpe agar	MRS	30	48	Thermo Fisher Scientific, Waltham, Massachusetts, USA
Modified LAB Selective	MLS	30	48	Manufactured as described by Xiao et al. 2024
Yeast Peptone Dextrose agar	YPD	30	48	Thermo Fisher Scientific, Waltham, Massachusetts, USA
Xylose Lysine Deoxycholate agar	XLD	37	16-20	SSI DIAGNOSTICA GROUP, Hillerød Denmark
Polymyxin-Acriflavin-Lithium-Ceftazidime-Aesculin-Mannitol agar	PAL	37	48	SSI DIAGNOSTICA GROUP, Hillerød Denmark
TSA+5% calf blood	BA	30	---	SSI DIAGNOSTICA GROUP, Hillerød Denmark
Buffered Peptone Water	BPW	37	24	Thermo Fisher Scientific, Waltham, Massachusetts, USA
Fraser	---	37	24	Thermo Fisher Scientific, Waltham, Massachusetts, USA
Half fraser	---	37	24	Thermo Fisher Scientific, Waltham, Massachusetts, USA
Lemuria Broth	LB	30	48	Thermo Fisher Scientific, Waltham, Massachusetts, USA

Table S2. Results of pairwise and overall PERMANOVA test comparing groups of each sampling month: 8.2023, 10.2023, 1.2024 and 4.2024**.** A) Results obtained from 16S rRNA data, B) results obtained from culture-dependent data.

A.

**Variable**	**Groups**		**DF**	**Sum of Sq**	**R^2**	**F**	**p-value**
Month	8.2023, 10.2023, 1.2024, 4.2024	**Model**	3	2.5469	0.65171	9.9797	1.00E-05
		**Residual**	16	1.3611	0.34829		
		**TOT**	19	3.9079	1		

**Group 1**	**Group 2**	**p-value**	**R^2**	**p (FDR)**
10.2023	8.2023	0.09618	0.1835571	0.09618
1.2024	8.2023	0.00799	0.7208565	0.01236
4.2024	8.2023	0.00824	0.4680572	0.01236
1.2024	10.2023	0.00784	0.7566077	0.01236
10.2023	4.2024	0.00771	0.4446842	0.01236
1.2024	4.2024	0.04102	0.3521694	0.049224

B.

**Variable**	**Groups**		**DF**	**Sum of Sq**	**R^2**	**F**	**p-value**
Month	8.2023, 10.2023, 1.2024, 4.2024	**Model**	3	0.0089	0.00297	0.0637	9.79E-01
		**Residual**	16	0.7457	0.04661		
		**TOT**	19	0.7546	1		

**Group 1**	**Group 2**	**p-value**	**R^2**	**p(FDR)**
10.2023	8.2023	0.17331	0.18765	0.17331
1.2024	8.2023	0.00805	0.42874	0.02415
4.2024	8.2023	0.05605	0.2636	0.1121
1.2024	10.2023	0.00805	0.33027	0.02415
10.2023	4.2024	0.0875	0.22157	0.13125
1.2024	4.2024	0.12814	0.23875	0.153768

Table S3. List of isolated microorganisms at species level identified by MALDI-TOF of the isolated bacteria from selective and not-selective agar media.

Species	8.2023	10.2023	1.2024	4.2024
Acinetobacter harbinensis	-	+	+	+
Aerococcus viridans	+	-	-	+
Aeromonas bestiarum	-	+	-	+
Aeromonas encheleia	-	+	+	-
Aeromonas eucrenophila	-	+	-	+
Aeromonas hydrophila	-	-	-	+
Aeromonas media	-	+	-	+
Aeromonas molluscorum	-	+	-	-
Aeromonas salmonicida	-	-	-	+
Aeromonas veronii	-	+	-	+
Aliivibrio finisterrensis	-	-	+	+
Aliivibrio logei	-	-	+	-
Aliivibrio sifiae	-	-	+	+
Bacillus cereus	+	-	+	+
Bacillus licheniformis	-	-	+	+
Bacillus mycoides	+	+	+	+
Bacillus pseudomycoides	-	-	+	-
Bacillus pumilus	-	-	+	+
Bacillus thuringiensis	-	-	-	+
Bacillus velezensis	-	-	+	-
Brevundimonas diminuta	-	+	+	-
Brochothrix thermosphacta	+	+	+	+
Buttiauxella ferragutiae	-	+	-	-
Candida zeylanoides	+	+	-	+
Carnobacterium maltaromaticum	+	+	-	+
Citrobacter braakii	+	+	-	-
Citrobacter freundii	-	-	+	-
Citrobacter gillenii	+	+	-	-
Corynebacterium ammoniagenes	+	-	-	-
Corynebacterium casei	+	-	-	-
Corynebacterium variabile	-	-	+	-
Debaryomyces hansenii	-	-	-	+
Enterobacter ludwigii	-	-	-	+
Enterococcus avium	+	-	-	-
Enterococcus devriesei	+	+	-	+
Enterococcus italicus	-	-	-	+
Enterococcus malodoratus	+	-	+	-

Enterococcus pseudoavium	+	-	+	+
Escherichia coli	-	+	-	+
Exiguobacterium marinum	-	+	-	-
Flavobacterium tegetincola	-	-	+	+
Gelidibacter mesophilus	+	-	-	-
Glutamicibacter bergerei	-	-	+	-
Klebsiella variicola	-	-	-	+
Kocuria rhizophila	-	+	-	-
Lactobacillus curvatus	-	-	+	-
Lactobacillus johnsonii	-	-	+	-
Lactococcus lactis	-	-	-	+
Latilactobacillus fuchuensis	-	+	+	-
Lelliottia amnigena	-	-	+	-
Micrococcus luteus	+	-	+	-
Morganella morganii	+	-	-	-
Myroides odoratus	-	+	-	-
Myroides phaeus	-	+	-	-
Pediococcus pentosaceus	-	-	-	+
Photobacterium iliopiscarium	-	-	-	+
Priestia megaterium	-	-	+	+
Proteus vulgaris	+	+	-	-
Providencia rettgeri	-	+	-	-
Providencia rustigianii	+	-	-	-
Pseudohyphozyma bogoriensis	-	-	-	+
Pseudomonas agarici	-	+	-	-
Pseudomonas anguilliseptica	-	-	+	+
Pseudomonas brenneri	-	-	+	-
Pseudomonas corrugata	-	+	+	-
Pseudomonas fluorescens	-	+	+	+
Pseudomonas fragi	+	-	+	+
Pseudomonas frederiksbergensis	-	-	-	+
Pseudomonas gessardii	-	+	+	+
Pseudomonas japonica	-	-	+	-
Pseudomonas kilonensis	-	+	-	-
Pseudomonas koreensis	-	-	+	-
Pseudomonas libanensis	-	+	-	-
Pseudomonas lundensis	-	-	-	+
Pseudomonas rhodesiae	-	+	+	+
Pseudomonas spp	-	-	+	-
Pseudomonas stutzeri	-	-	+	-
Pseudomonas synxantha	-	-	+	-

Pseudomonas taetrolens	-	+	+	+
Pseudomonas veronii	-	+	-	-
Psychrobacter aquaticus	+	-	+	-
Psychrobacter immobilis	+	+	+	+
Psychrobacter luti	-	-	+	+
Psychrobacter maritimus	+	+	+	+
Psychrobacter pasteurii	-	+	-	-
Psychrobacter sanguinis	-	+	-	-
Psychrobacter vallis	-	+	+	-
Raoultella ornithinolytica	-	-	-	+
Raoultella planticola	-	+	-	-
Raoultella terrigena	-	-	-	+
Rhodococcus erythropolis	-	+	+	-
Rhodotorula mucilaginosa	+	+	-	-
Rothia terrae	+	-	-	-
Shewanella algae	-	+	-	-
Shewanella baltica	+	+	+	+
Shewanella colwelliana	+	-	+	-
Shewanella frigidimarina	-	-	+	+
Shewanella gaetbuli	-	+	-	-
Shewanella hafniensis	-	-	-	+
Shewanella hanedai	-	-	-	+
Shewanella indica	+	+	-	-
Shewanella putrefaciens	-	+	+	+
Staphylococcus capitis	-	-	+	-
Staphylococcus epidermidis	+	-	+	-
Staphylococcus equorum	-	+	-	-
Staphylococcus haemolyticus	+	-	-	-
Staphylococcus hominis	+	-	-	+
Staphylococcus pasteuri	-	+	+	-
Staphylococcus sciuri	-	+	-	-
Staphylococcus simulans	+	-	+	-
Staphylococcus warneri	+	+	+	+
Streptococcus lutetiensis	-	-	+	-
Streptomyces flavotricini	-	-	+	-
Streptomyces roseolus	-	-	+	-
Streptomyces virginiae	-	-	+	-
Vagococcus fluvialis	+	-	-	-
Vibrio alginolyticus	+	-	-	-
Vibrio xuii	-	-	+	+
Weizmannia ginsengihumi	-	-	-	+

Yarrowia deformans	-	+	-	-
Yarrowia lipolytica	-	+	+	+

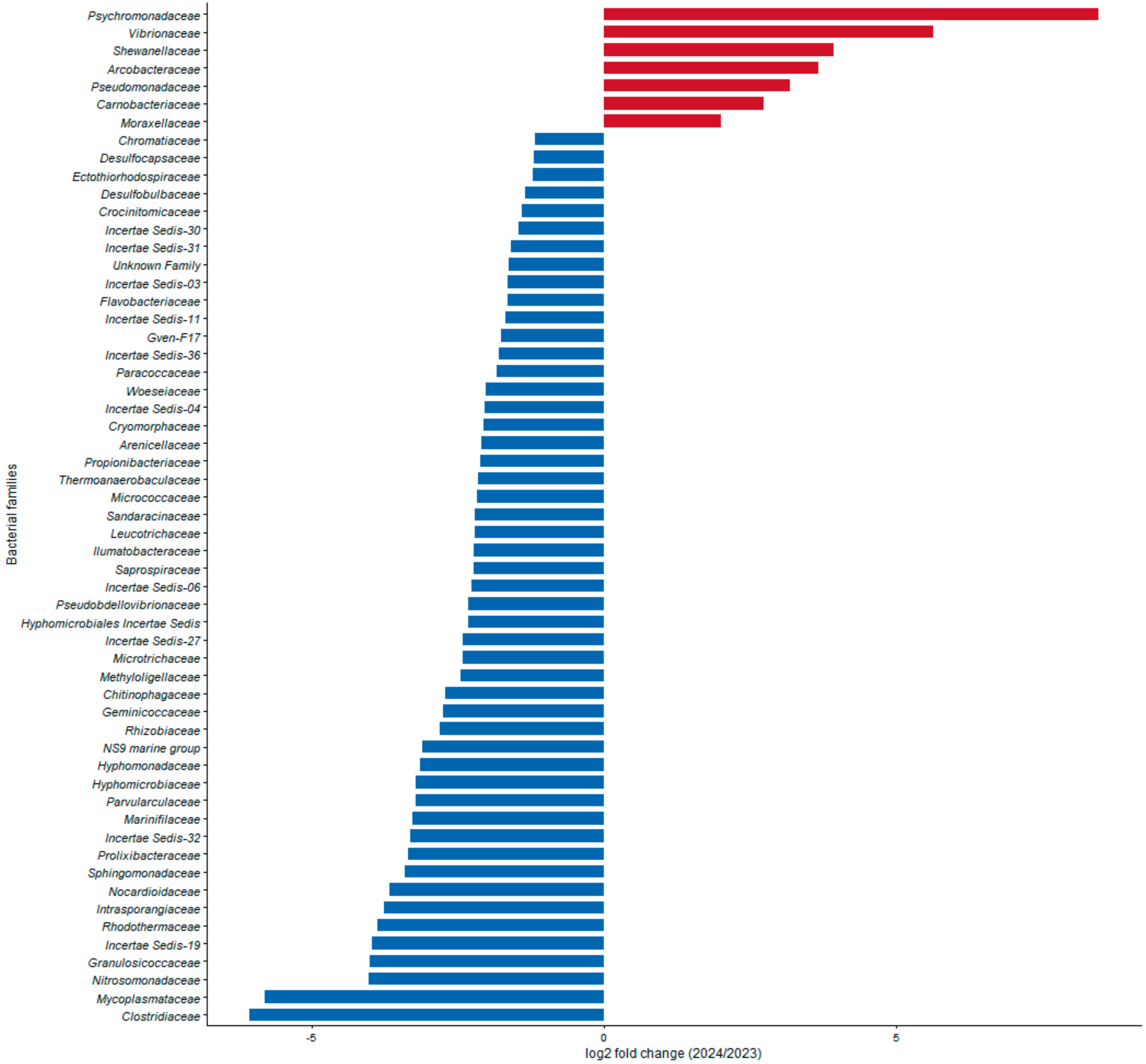
Psychromonadaceae
Vibrionaceae
Shewanellaceae
Arcobacteraceae
Pseudomonadaceae
Carnobacteriaceae
Moraxellaceae
Chromatiaceae
Desulfocapsaceae
Ectothiorhodospiraceae
Desulfobulbaceae
Crocinitomicaceae
Incertae Sedis-30
Incertae Sedis-31
Unknown Family
Incertae Sedis-03
Flavobacteriaceae
Incertae Sedis-11
Gven-F17
Incertae Sedis-36
Paracoccaceae
Woeseiaceae
Incertae Sedis-04
Cryomorphaceae
Arenicellaceae
Propionibacteriaceae
Thermoanaerobaculaceae
Micrococcaceae
Sandaracinaceae
Leucotrichaceae
Ilumatobacteraceae
Saprospiraceae
Incertae Sedis-06
Pseudobdellovibrionaceae
Hyphomicrobiales Incertae Sedis
Incertae Sedis-27
Microtrichaceae
Methyloligellaceae
Chitinophagaceae
Geminicoccaceae
Rhizobiaceae
NS9 marine group
Hyphomonadaceae
Hyphomicrobiaceae
Parvularculaceae
Marinifilaceae
Incertae Sedis-32
Prolixibacteraceae
Sphingomonadaceae
Nocardioidaceae
Intrasporangiaceae
Rhodothermaceae
Incertae Sedis-19
Granulosicoccaceae
Nitrosomonadaceae
Mycoplasmataceae
Clostridiaceae
Bacterial families
-5
0
5
log2 fold change (2024/2023)

Figure S1. Bar plot showing bacterial families exhibiting half-season differential abundance between 2023 and 2024. Bars represent $\log_2$ fold change for bacterial families displaying both large effect size ($|\log_2| \geq 1$) and statistically significant differences ($p < 0.05$). Negative fold changes are highlighted in blue and positive in red.

.
